# Prognostic value of c-Met overexpression in hepatocellular carcinoma: a meta-analysis and review

**DOI:** 10.18632/oncotarget.20087

**Published:** 2017-08-09

**Authors:** Jung Han Kim, Hyeong Su Kim, Bum Jun Kim, Hyun Joo Jang, Jin Lee

**Affiliations:** ^1^ Division of Hemato-Oncology, Department of Internal Medicine, Kangnam Sacred-Heart Hospital, Hallym University Medical Center, Hallym University College of Medicine, Seoul 07441, Republic of Korea; ^2^ Division of Gastroenterology, Department of Internal Medicine, Dongtan Sacred-Heart Hospital, Hallym University Medical Center, Hallym University College of Medicine, Hwasung 18450, Republic of Korea; ^3^ Department of Internal Medicine, National Army Capital Hospital, The Armed Forces Medical Command, Sungnam 13574, Republic of Korea

**Keywords:** c-Met, hepatocellular carcinoma, prognostic value, meta-analysis

## Abstract

The overexpression of c-Met protein has been detected in hepatocellular carcinoma (HCC). However, its prognostic impact remains uncertain. We performed this meta-analysis to evaluate the prognostic value of c-Met overexpression in patients who underwent curative surgical resection for HCC. A systematic computerized search of the electronic databases was carried out. From 5 studies, 1,408 patients who underwent surgical resection for HCC were included in the meta-analysis. Compared with patients with HCC having low c-Met expression, patients with c-Met-high tumor showed significantly worse relapse-free survival (hazard ratio = 1.26 [95% confidence interval, 1.02–1.56], *P* = 0.03) and overall survival (hazard ratio = 1.16 [95% confidence interval, 1.03–1.31], *P* = 0.01). In conclusion, our meta-analysis indicates that c-Met overexpression is a significant adverse prognostic factor for recurrence and survival in patients who underwent surgical resection for HCC.

## INTRODUCTION

Hepatocellular carcinoma (HCC) is the fifth most common cancer worldwide [1]. Despite the recent advances in diagnostic and therapeutic modalities, HCC is still one of the most lethal malignancies [2, 3]. Surgical resection is the first choice of treatment for patients with HCC at early stage, but it is possible only in a small proportion of patients because of impairment of liver function caused by underlying cirrhosis or advanced disease at the time of diagnosis [4, 5]. Moreover, more than half of the patients who underwent complete resection eventually develop recurrent diseases or *de novo* tumors during the course of their disease [6].

For patients with advanced HCC, systemic treatment with sorafenib or sunitinib (oral, small-molecule, multi-targeted receptor tyrosine kinase inhibitors targeting receptors for platelet-derived growth factor and vascular endothelial growth factor) can be recommended [7, 8]. However, their survival benefits are disappointing, and thus, new effective treatments are still required. c-Met has recently emerged as a possible therapeutic target in various tumors including HCC and some drugs targeting the c-Met signaling pathway are under investigation in clinical trials [9].

c-Met is the product of the proto-oncogene *MET* and the tyrosine kinase receptor for hepatocyte growth factor (HGF) [10]. HGF, also known as a scatter factor, binds to c-Met and initiates auto-phosphorylation of multiple tyrosine residues in the intracellular region. The c-Met/HGF signaling pathway regulates multiple cellular functions, including differentiation, proliferation, and angiogenesis [11, 12]. c-Met also plays critical roles in the pathogenesis of cancer. It is implicated in the molecular mechanisms of tumor cell proliferation, survival, invasion, and metastasis [13]. The enhanced expression of c-Met has been observed in various tumors, such as breast cancer [14], lung cancer [15], gastric cancer [16], colorectal cancer [17], cervix cancer [18], or pancreatic cancer [19]. Several meta-analyses in common tumors indicated that high c-Met expression was associated with a poor prognosis [14–18].

The overexpression of c-Met has also been observed in HCC [20–28]. However, its prognostic impact has not been consistent among studies. Therefore, we performed this meta-analysis to evaluate the prognostic value of c-Met overexpression in patients who underwent curative surgical resection for HCC. To our knowledge, this is the first meta-analysis regarding the prognostic impact of c-Met overexpression in patients with HCC.

## RESULTS

### Results of search

**Figure 1:**
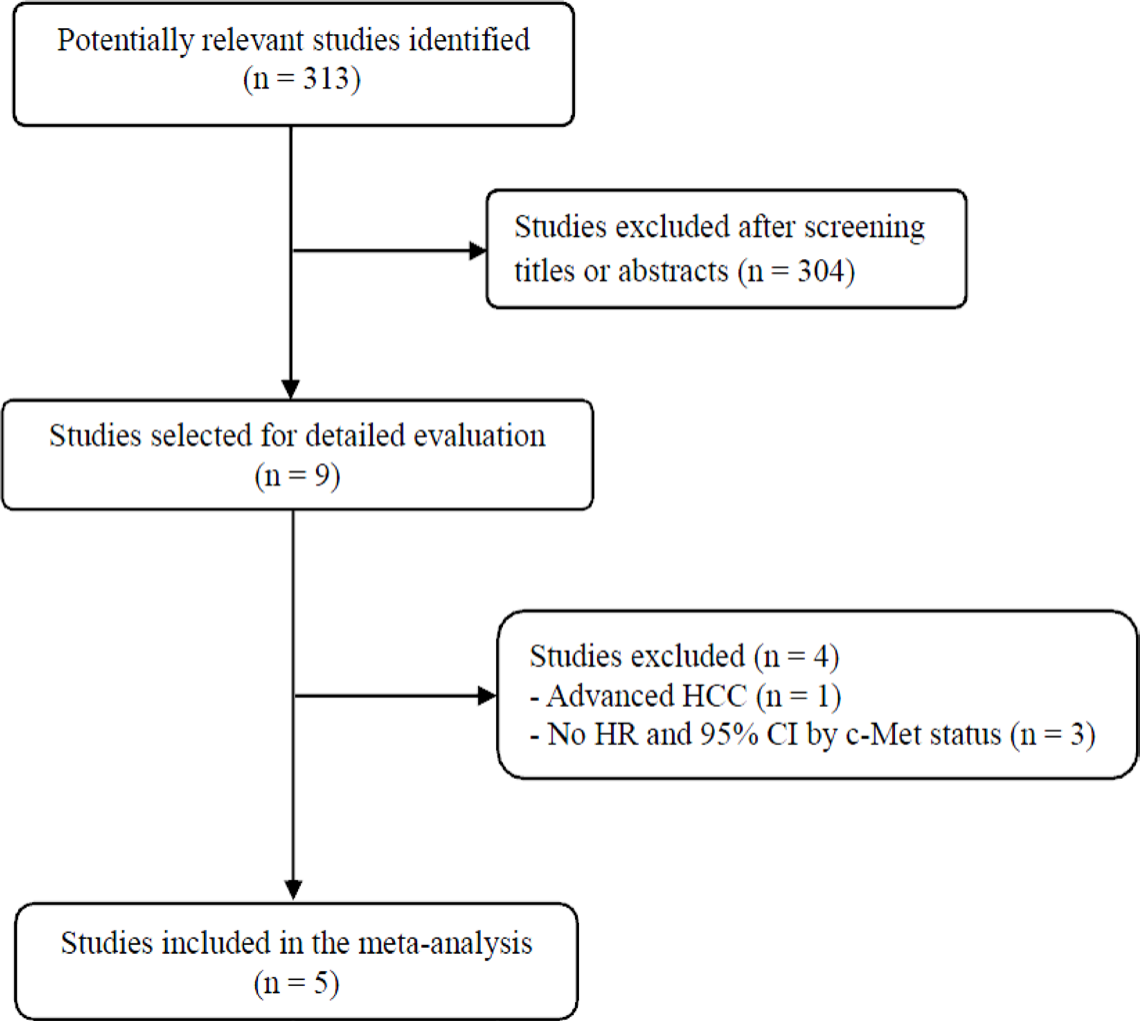
Flow diagram of search process

Figure 1 shows the flowchart of our study. A total of 313 potentially relevant studies were initially found, but 304 of them were excluded after screening the titles and abstracts. Of the remaining 9 potentially eligible studies, 4 were further excluded by the inclusion criteria: one was conducted in advanced HCC [20] and three had no data from which the required hazard ratio (HR) with 95% confidence interval (CI) stratified by the c-Met status (low or high) could be extracted [21–23]. Finally, 5 studies were included in the meta-analysis [24–28].

### Characteristics of the included studies

Table 1 summarizes the main characteristics and clinical outcomes of the five included studies. All the studies were performed retrospectively in HCC patients who underwent curative surgical resection. From the 5 studies, 1,408 patients were included in the meta-analysis. One study used Western blot to assess the c-Met status [24], and the remaining 4 used immunohistochemistry (IHC).

**Table 1:** Summary of the five included studies

					c-Met results			
**Author (year) Country**	**Methods**	**Antibody, Dilution**	**No. of patients**	**Criteria for c-Met^high^**	**c-Met ^low^**	**c-Met ^high^**	**HR for RFS (95% CI)**	**HR for OS (95% CI)**
Ueki *et al.*, (1997) Japan	Western blot	Not applicable	62	≥ Cutoff point of median value (36.4%), compared with the Hep3B band	32 (51.6%)	30 (48.4%)	NA	2.26 (1.00–5.11) *P* = 0.051
Ke *et al*., (2008) China	IHC	Anti-human c-Met, EPI1454Y, 1:100	520	> 20% of tumor section	238 (45.85%)	282 (54.2%)	1.18 (0.95–1.46) *P* = 0.111	1.23 (0.97–1.53) *P* = 0.118
Lee *et al.,* (2013) Korea	IHC	Rabbit monoclonal anti-c-Met, 1:100	287	Proportion of stained tumor cells: 0 = < 20%; 1 = 20–60%; 2 = 61–80%; 3 = ≥ 81% (c-Met^high^: 2 or 3)	207 (72.1%)	80 (27.9%)	1.099 (0.86–1.41) *P* = 0.461	1.095 (0.92–1.30) *P* = 0.299
Kondo *et al.*, (2013) Japan	IHC	Rabbit polyclonal anti-c-Met, 1:500	59	Membrane staining: 0 = no; 1 = weak and incomplete or weak but complete < 10% of tumor cells; 2 = weak but complete in ≥ 10% or intense and complete circumferential staining in < 30%; 3 = intense and complete in ≥ 30% (c-Met^high^: 2 or 3)	44 (74.6%)	15 (25.4%)	3.10 (1.49–6.46) *P* = 0.002	0.96 (0.44–2.07) *P* = 0.91
Koh *et al.,* (2015) Korea	IHC	Rabbit polyclonal anti-c-Met, 1:50	490	Reactivity/membrane or cytoplasmic staining; 0 = no reactivity; 1= weak/faint or light; 2 = moderate/ intermediate in at least 30% or tumor cells; 3 = strong/intense complete in ≥ 30% (c-Met^high^: 2 or 3)	190 (38.8%)	300 (61.2%)	1.25 (1.01–1.55) *P* = 0.046	1.21 (0.91–1.60) *P* = 0.199

IHC, immunohistochemistry; RFS, relapse-free survival; OS, overall survival; HR, hazard ratio; CI, confidence interval; NA, not available.

### c-Met expression status

There was a marked heterogeneity between the criteria used to dichotomize c-Met status (c-Met^low^ or c-Met^high^). The criteria are briefly summarized in the Table 1. The rate of high c-Met expression ranged from 25.4% [27] to 61.2% [28].

### Impact of c-Met expression on relapse-free survival

**Figure 2:**
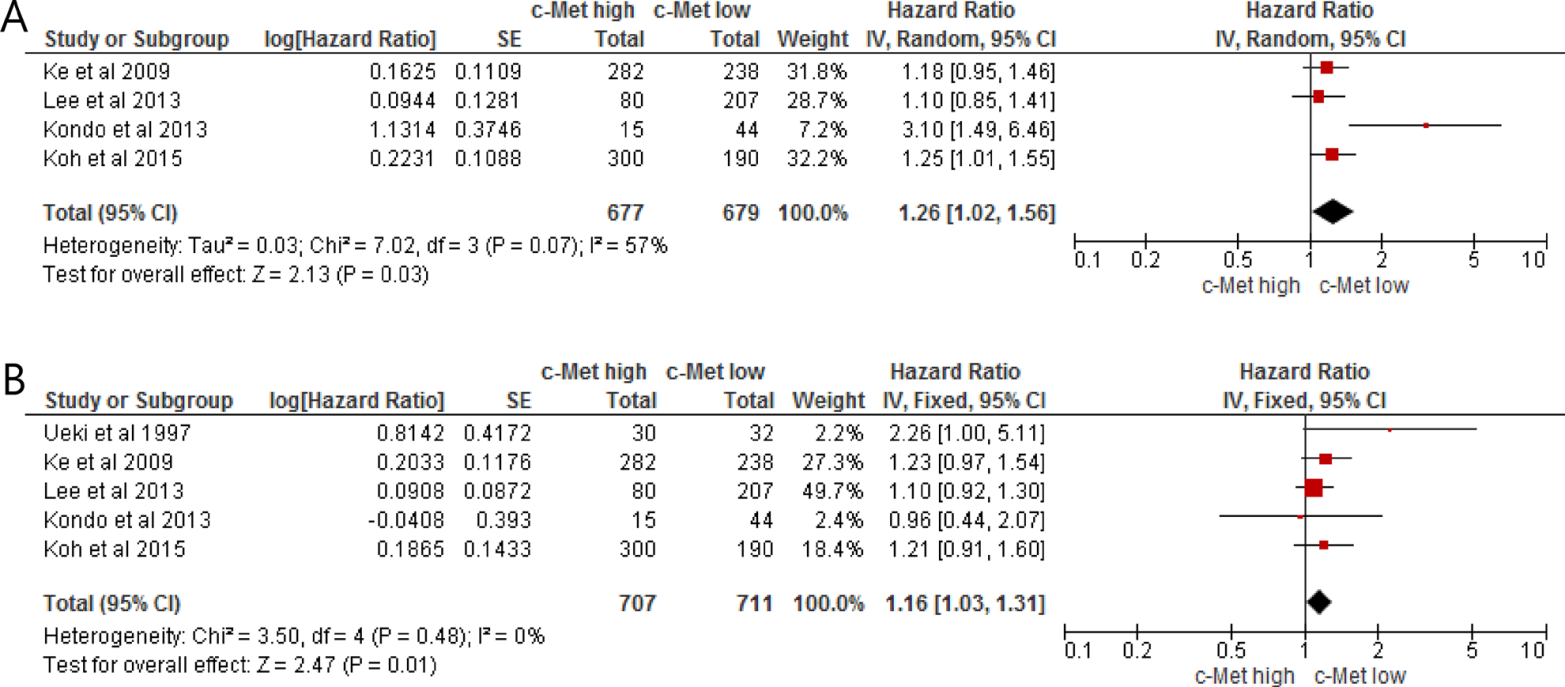
Forest plots of hazard ratios for relapse-free survival (**A**) and overall survival (**B**).

From three studies [24–28], 1,356 patients were included in the meta-analysis of HRs for relapse-free survival (RFS). Compared with HCC patients with low c-Met expression, patients with c-Met-high HCC showed significantly worse RFS (HR = 1.26 [95% CI, 1.02–1.56], *P* = 0.03) (Figure 2A). There was a significant heterogeneity among studies (*X*^2^ = 7.02, *P* = 0.07, *I*^2^ = 57%) and the random-effect model was selected.

### Impact of c-Met expression on overall survival

From five studies [24–28], 1,408 patients were included in the meta-analysis of HRs for OS. Patients with c-Met-high HCC showed significantly worse OS than those with c-Met-low tumor (HR = 1.16 [95% CI, 1.03–1.31], *P* = 0.01) (Figure 2B). The fixed-effect model was applied because there was no significant heterogeneity across the studies (*X*^2^ = 3.50, *P* = 0.48, *I^2^* = 0%).

### Publication bias

**Figure 3:**
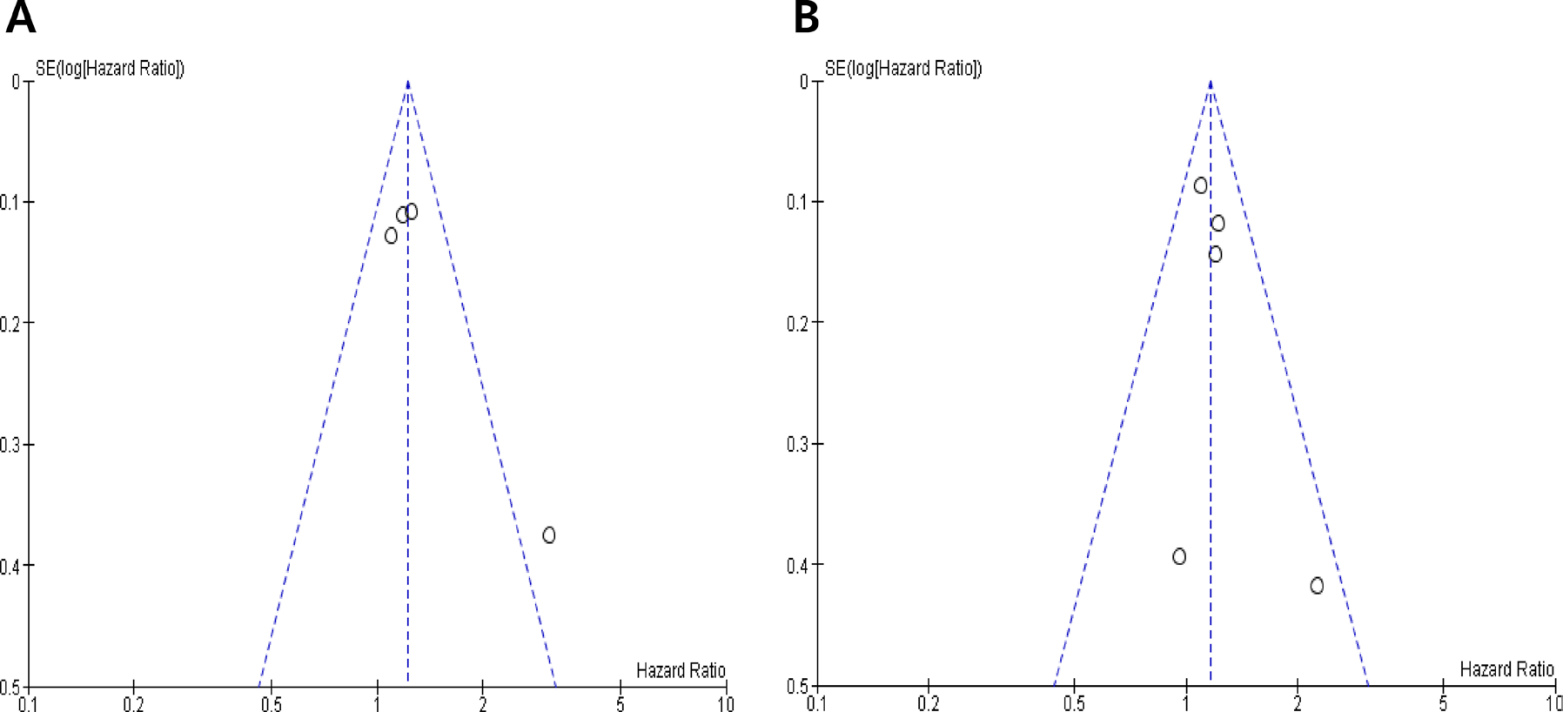
Funnel plots for publication bias regarding relapse-free survival (**A**) and overall survival (**B**).

Visual inspection of the funnel plots for RFS and OS showed symmetry, indicating there were no substantial publication biases (Figure 3A and 3B).

## DISCUSSION

In this meta-analysis, we evaluated the prognostic impact of c-Met overexpression in patients with surgically resected HCC. The results show that high c-Met expression is significantly associated with worse RFS and OS. Our findings suggest that c-Met overexpression represent a potential adverse prognostic marker in patients who received curative surgery for HCC.

c-Met plays a critical role in the pathogenesis and progression of many tumor types [10–13]. The enhanced expression of c-Met has also been detected in HCC [20–30]. Different molecular alterations have been found to determine c-Met activation: point mutations, gene amplifications, enhanced transcription, and autocrine activation [31]. In HCC, the aberrant activation of c-Met signaling results mainly from its overexpression due to enhanced transcription, rather than from gene mutations or amplifications [32]. The previous studies have suggested that c-Met overexpression is significantly associated with clinicopathological features of HCC, such as tumor grade [29], vascular invasion or thrombosis [23], tumor recurrence [30], metastases [27, 30], and worse RFS [27, 28] or OS [22–24]. A recent retrospective study with 194 HCC patients treated by hepatic resection or microwave ablation found that c-Met overexpression was associated with unfavorable survival outcomes [22]. However, these results are contrary to the findings reported in other studies, in particular with respect to OS [26–28]. Recently, Lee *et al.* assessed c-Met expression and amplification in 287 patients with HCC and reported that c-Met overexpression was not significantly associated with any clinicopathological variable including tumor grade and size, vascular invasion, stage, RFS, and OS [26]. Because many studies had a small number of patients and adopted various methods and criteria for c-Met expression status [20–28], however, they could not draw a consensus regarding the prognostic value of c-Met expression.

In the current meta-analysis, we included studies comparing survival outcomes (RFS or OS) according to c-Met expression status (low vs. high). Patients with c-Met-high HCC showed significantly worse RFS (HR = 1.26, *P* = 0.03) and OS (HR = 1.16, *P* = 0.01), compared with those with c-Met-low HCC. Our results indicate that high c-Met expression is an independent negative prognostic marker for recurrence and survival in HCC patients who received curative surgical resection.

Several meta-analyses in other cancers have also demonstrated that high expression of c-Met is an adverse prognostic marker [14–17]. Thus, interference with c-Met activation may provide an effective therapeutic strategy for cancers with c-Met overexpression [33]. Based on the therapeutic rationale to target c-Met, various c-Met inhibitors are currently under active investigation in a variety of cancers, including HCC [9, 34–37]. Tivantinib, an oral selective c-Met receptor tyrosine kinase inhibitor, showed promising results as a second-line treatment in a randomized phase II trial of advanced HCC [36]. Interestingly, in the post hoc analysis of the c-Met high subgroup, patients treated with tivantinib showed better time-to-progression (median 2.7 vs. 1.4 months, HR = 0.43, *P* = 0.03) and OS (median 7.2 vs. 3.8 months, HR = 0.38, *P* = 0.01), compared with those treated with placebo. Thus, patients with HCC overexpressing c-Met might be good candidates for treatment with c-Met inhibitors. In addition, in a recent randomized phase III trial of patients with advanced non-small-cell lung cancer, the efficacy of tivatinib in combination with erlotinib was also significantly associated with c-Met expression [34]. These results suggest the importance of indentifying predictive biomarkers for benefits in drug development.

However, the major limitation for development of c-Met inhibitors is that there is no consensus regarding the criteria for c-Met overexpression. Currently, a variety of methods (IHC, Western blot, fluorescence *in situ* hybridization, real-time quantitative PCR, or molecular invasion probe, etc) are used for assessing c-Met status, with no standardized criteria for overexpression. In addition, there are differences in the IHC criteria for high c-Met expression. The discrepancies in the prognostic impact of c-Met overexpression among studies might be attributable to the different methods and criteria for c-Met overexpression. Therefore, the definition of reliable criteria for Met-high status is essential to identify patients who will benefit most from MET-targeted therapies.

Our study has several inherent limitations that need to be noted. First, the meta-analysis included the small number of studies. Second, all the studies were retrospectively performed. Third, the five studies were all conducted in Asia. However, it is unlikely that there is a significant difference in the prognostic impacts of c-Met in HCC between Asia and Western countries. Forth, the studies used different methods (Western blot or IHC) and criteria to assess and stratify c-Met status, which might lead to the wide variation in the rate of c-Met overexpression among studies. Finally, papers published only in English were included, which might bias the results.

In conclusion, our meta-analysis demonstrates that c-Met overexpression is a significant adverse prognostic marker for recurrence and survival in patients who underwent surgical resection for HCC. However, larger prospective studies using standardized methods are still needed to verify the prognostic role of c-Met expression.

## MATERIALS AND METHODS

### Publication searching strategy

We performed this study according to the Preferred Reporting Items for Systematic Reviews and Meta-Analyses (PRISMA) guidelines [38]. A systematic computerized search of the electronic databases PubMed, Embase, and Google scholar (up to May 2017) was carried out. The search used the following keywords: ‘c-Met’ or ‘Met’ and ‘hepatocellular carcinoma’ or ‘hepatoma’ or ‘liver neoplasm.’ The related articles function in the PubMed was also used to identify all related articles.

### Inclusion criteria

Eligible studies should meet the following inclusion criteria: (i) patients had a diagnosis of HCC and underwent curative surgical resection; (ii) RFS and/or OS were analyzed according to the c-Met expression status (low vs. high); (iii) HRs with 95% CIs for RFS or OS were reported or could be estimated from the data provided; (iv) articles were written in English.

### Data extraction

Data extraction was carried out independently by two investigators (BJK and HSK). If these two authors did not agree, the principle investigator (JHK) was consulted to resolve the dispute.

The following data were extracted from all eligible studies: first author's name, year of publication, country, number of patients, treatment, methodology for c-Met expression, the criteria used to dichotomize c-Met expression as ‘high’ or ‘low’, and HRs with their 95% CIs for RFS or OS.

### Statistical analysis

Statistical values were obtained directly from the original articles. When HR with its 95% CIs was not provided, the Engauge Digitizer version 9.1 was used to estimate the needed data from Kaplan-Meier curves. The effect size of RFS and OS was pooled through HR and its 95% CI. The heterogeneity across studies was examined by *Q* statistic and the *I*^2^ inconsistency test. The fixed-effect model (Mantel–Haenszel method) was selected for pooling homogeneous outcomes when *P* ≥ 0.1 and *I*^2^ *≤* 50%, and the random-effects model (DerSimonian–Laird method) was applied for pooling heterogeneous outcomes when *P* < 0.1 and *I*^2^ > 50%. The RevMan software version 5.2 was used to combine the data, and the final results were presented with HRs and their 95% CIs. All reported *P*-values were two-sided and *P* < 0.05 was considered statistically significant. Publication bias was assessed graphically by the funnel plot method [39].
